# Antileishmanial Activity of Compounds Derived from the Medicines for Malaria Venture Open Access Box against Intracellular *Leishmania major* Amastigotes

**DOI:** 10.4269/ajtmh.15-0448

**Published:** 2016-02-03

**Authors:** Mozna Khraiwesh, Susan Leed, Norma Roncal, Jacob Johnson, Richard Sciotti, Philip Smith, Lisa Read, Robert Paris, Thomas Hudson, Mark Hickman, Max Grogl

**Affiliations:** Division of Experimental Therapeutics, Walter Reed Army Institute of Research, Silver Spring, Maryland; Department of Clinical Investigation, Madigan Army Medical Center, Tacoma, Washington; Military Malaria Research Program, Walter Reed Army Institute of Research, Silver Spring, Maryland

## Abstract

Leishmaniasis is a complex tropical disease caused by kinetoplastid parasitic protozoa of the genus *Leishmania* and is transmitted by the sand fly insect vector. Cutaneous leishmaniasis (CL) is the most common form of this disease, and CL infections often result in serious skin lesions and scars. CL remains a public health problem in many endemic countries worldwide because of the absence of effective, safe, and cost-effective drugs for treatment. One of the strategies we chose to use to find novel chemical entities worthy of further development as antileishmanials involved screening synthetic and natural products libraries. In our study, we developed a *Leishmania major* intracellular amastigote assay that uses the activity of luciferase as a measure of parasite proliferation and used this assay to screen a collection of 400 compounds obtained from Medicines for Malaria Venture (MMV) for their antileishmanial activity. Our results showed that 14 compounds identified by MMV as antimalarial drugs have antileishmanial activity and can potentially be optimized for CL drug development.

## Introduction

Leishmaniasis is a vector-borne disease caused by parasites of the genus *Leishmania*. Leishmaniasis is endemic in the tropics, subtropics, and the Mediterranean basin, with more than 1.5 million estimated new cases per year and an additional 350 million people at risk of infection.^1^ The clinical spectrum of leishmaniasis ranges from self-healing cutaneous ulcers to irreparable damage of soft and cartilaginous tissues and even to fatal systemic illness.^2^

The clinical manifestations of cutaneous leishmaniasis (CL) encompass a wide spectrum of severity and present in a range of clinical forms.^3^ Because of lack of an effective vaccine and inadequate therapy, CL remains a public health problem in many endemic countries worldwide. CL is not a life-threatening condition, and severe complications are infrequent. Many different therapeutic interventions, including topical, systemic, and nonpharmacological treatments, have been described. At this time, the drugs used for CL include older drugs such as pentavalent antimony, pentamidine, amphotericin B, and newer drugs such as the imidazoles, miltefosine, paromomycin, and liposomal amphotericin B. All of these drugs have potential issues associated with them, including toxicity, emerging resistance, parenteral administration, high cost, and relatively long treatment regimens. The treatment decision should be based first on the risk-benefit ratio of the intervention for each patient. In addition, it has been shown that the infecting species and geographical region affect the efficacy of treatments and therefore treatment recommendations. Thus, the CL physician toolbox requires multiple tools, since there is no one drug that is the ideal answer for all cases. The overall need for the treatment of CL infections that do not rapidly self-cure, cause potentially disfiguring or disabling lesion(s), or the potential risk of the infecting strain(s) causing metastasis is high. Therefore, beside a safe and effective topical formulation for uncomplicated CL, an effective, well-tolerated, orally bioavailable agent is needed. This oral agent needs to be active against several etiologic species, yield superior cosmetic results, adapted for use in rural areas, and must be manufactured at low cost.^4^

Various strategies were adopted to search for new treatments against leishmaniasis, including rational design of drugs,^5^,^6^ screening synthetic and natural products libraries.^7^ The Medicines for Malaria Venture (MMV) Box has been a tool for discovering drugs for neglected tropical diseases.^8^ The Malaria Box is a set composed of 400 diverse compounds derived from 20,000 antimalarial hits generated by screening corporate and academic libraries.^9^–^11^ We hypothesized that some of these antimalarial hits could serve as a starting point to accelerate drug discovery for other neglected diseases.^12^ In close collaboration between The Drug for Neglected Diseases Initiative (DND*i*) and the University of Antwerp, all compounds in MMV Box were screened against parasites responsible for human African trypanosomiasis, visceral leishmaniasis, and Chagas disease.^13^ On November 13, 2012, DND*i* and MMV announced the identification of two potential drug series for sleeping sickness treatment and one for visceral leishmaniasis.^13^

Assessments of the susceptibility of the amastigote-macrophage *Leishmania* parasite form to antileishmanial drugs has been classically performed by visual examination and counting of stained intracellular amastigotes on glass microscopic slides; however, this method of amastigote-macrophage quantitation is labor intensive and does not provide the throughput required to screen large numbers of compounds.^14^ In the past few years, reporter gene technology has been developed to make drug screening more efficient by producing objective quantitative data, decreasing manual labor, and increasing throughput.^14^,^15^ In addition, new high-content screens utilizing reporter proteins and fluorescent dyes have been established for conducting amastigote-macrophage dose–response and screen assays.^16^–^18^

Primary screening assays used for drug discovery campaigns have typically used free-living forms of the *Leishmania* parasite to allow for high-throughput screening (HTS). Unfortunately, such screens do not necessarily reflect the physiological situation, as amastigotes, the disease-causing stage of the parasite, are intracellular and not free-living parasites. An alternative is to use axenic amastigotes;^19^–^21^ however, several reports indicate that axenic amastigotes are different from intracellular amastigotes both in terms of drug susceptibility and protein expression.^22^–^24^ One strategy that has been used for some screens is initial promastigote HTS testing followed by dose–response testing of hits against the amastigote-macrophage form of the parasite.^18^ Nevertheless, recently with the advent of new technologies, it has become possible to increase the throughput of the traditionally very labor-intensive intracellular amastigotes assays. To our knowledge, two main methods are in use for the detection of intracellular amastigotes: plate reader–based methods that rely on reporter constructs and microscopy-based methods that count parasites directly.^16^,^18^,^25^,^26^

In this study, we screened a collection of compounds from MMV using both *Leishmania major* prescreen and dose–response assays. Our intracellular amastigote assay is a microtiter plate drug sensitivity assay that uses the activity of luciferase as a measure of the proliferation of luciferase-expressing *L*. *major* parasites developing intracellularly inside RAW 264.7 macrophages in the presence of antileishmanial drugs or experimental compounds. The hits derived from this assay were further tested for toxicity against the RAW 264.7 macrophage line to weed out compounds with toxicity not associated with antileishmanial activity. The final results from this screen were further examined for their potential development as antileishmanial drugs. The development of cheap, orally administered, novel antileishmanial drugs with activity against various species will require intensive screening of novel chemical entities to find candidate compounds worthy of further development. As CL is one of the neglected diseases, many screening campaigns such as this will be necessary to find compounds that one day are approved and in use by the poorest of the poor affected by this neglected disease.

## Materials and Methods

### Compounds.

Amphotericin B, miltefosine, and paromomycin were purchased from Sigma-Aldrich (St. Louis, MO). Pentostam was obtained from Calbiochem (EMD Millipore, Billericca, MA). Sitamaquine and pentamidine were obtained from the Walter Reed Army Institute of Research Chemical Repository. The Malaria Box, a set of 400 diverse compounds assembled by MMV, was acquired from MMV. This collection consists of 200 diverse drug-like compounds suitable for oral administration and 200 diverse probe-like compounds for use as biological tools for drug discovery and development for neglected diseases. This collection was distilled from 20,000 hits generated by an extensive screening campaign of 4 million compounds conducted by St. Judes Children's Research Hospital, Novartis, and Glaxo-Smith Kline.^9^–^12^ The selection was made to provide the broadest cross section of structural diversity and, in the case of the drug-like compounds, properties commensurate with oral absorption and the minimum presence of toxicophores.

### Cell culture.

*Leishmania major* (strain code MHOM/SU/73/WR779) promastigotes were maintained in Schneider's media (Lonza, Walkersville, MD) supplemented with 10% heat-inactivated fetal bovine serum (Invitrogen, Grand Island, NY) at 22.5°C. RAW 264.7 (ATCC, Manassas, VA) macrophages were maintained in Dulbecco's modified eagle's medium (DMEM) (catalog no. 30-2002, ATCC) supplemented with 20% heat-inactivated fetal bovine serum at 37°C incubator supplied with 5% CO_2_.

### Production of the luciferase-expressing *L*. *major* parasite.

The luciferase expression construct was created in the same manner as shown by Lecoeur and others^27^ by digesting the luciferase coding region (1.66 kbp) of pGL3-Basic (Promega, Madison, WI) by using two restriction enzymes, NcoI/EagI, followed by electrophoretic separation of the luciferase coding region on a 1% agarose gel. The luciferase coding region was then ligated into pLEXSY-hyg2 vector (Jena Biosciences, Jena, Germany), which had previously been digested with NcoI/NotI. The vector was linearized with SwaI and subsequently gel purified before transfection into *L*. *major* parasites. Transfections were carried out by electroporation at 480 V, 13 Ω, and 500 μF (0.4 mL of 1 × 10^8^ parasites/mL, and 0.1 mL of 10 μg DNA). Cuvettes were placed on ice before electroporation. On the completion of electroporation, cuvettes were cooled on ice for 10 minutes. Parasites were returned to Schneider's media containing penicillin/streptomycin and gentamycin for 24 hours. Selection for transfectants was then carried out using hygromycin B (100 μg/mL) after 24 hours of transfection.

### Luminescence assay.

#### Dose–response determinations.

Macrophages were harvested from culture by removing all spent media, adding in 10 mL of fresh media, scraping adherent macrophages from the culture flask, and counting macrophages using trypan blue. The cells were resuspended at 2.0 × 10^5^ cells/mL, and then dispensed in a volume of 50 μL to yield a final concentration of 10,000 macrophages/well in 384-well tissue culture–treated sterile white plates (Nunc/Thermo Fisher, Rochester, NY) using a Tecan EVO Freedom robotics system (Tecan U.S., Durham, NC). The plates were then incubated at 37°C in 5% CO_2_ atmosphere for 24 hours. After incubation, the media was removed from each well using the Tecan EVO Freedom robot, and 1 × 10^5^ transfected *L*. *major* promastigotes were added to each well in a volume of 50 μL in DMEM/heat-inactivated fetal bovine serum (HIFBS) media and allowed to invade the macrophages. After overnight incubation, the media was removed from each well using the Tecan Freedom EVO robot, and each well was subsequently washed three times with 40 μL of fresh DMEM/HIFBS medium to remove all extracellular promastigotes. After the third wash, 69.2 μL of DMEM/HIFBS medium was added to each well using the Tecan EVO Freedom robot. Drug plates were prepared with the Tecan EVO Freedom using sterile 96-well plates containing 12 duplicate 2-fold serial dilutions of each test compound suspended in dimethyl sulfoxide (DMSO) for dose–response determinations. Of diluted test compound, 7.8 μL was added to the 69.2 μL media present in each well providing a 10-fold final dilution of compound. The final concentration range tested was 0.0005–1 μM for all assays. The plates were placed in a modular incubator chamber (Billups-Rothenburg, Del Mar, CA) that contained a humidity source, consisting of soaked gauze in a weigh boat, which was placed in the bottom of the chamber. Both valves on the system were opened and using a vent filter (Millipore, Billerica, MA), attached to the one valve, mixed gas containing 5% O_2_, 5% CO_2_, and a balance of nitrogen was allowed to pass through the system for 60 seconds. After 60 seconds, the exit valve was closed and the mixed gas was allowed to flow into the chamber for 10 seconds. At that point the remaining valve was closed. Chambers were gassed every other day. The chambers were incubated at 37°C for 96 hours. After 96 hours of incubation, 7.5 μL luciferin solution (Caliper Life Science, Hopkinton, MA) diluted to 150 μg/mL was added to each well, and the plates were incubated for 30 minutes at 37°C in the dark. Each plate was read using an Infinite M200 plate reader (Tecan Inc., Durham, NC).

The 50% inhibitory concentrations (IC_50_) were then generated for each dose–response test using GraphPad Prism (GraphPad Software Inc., San Diego, CA) using the nonlinear regression (sigmoidal dose–response/variable slope) equation.

#### Prescreen assay.

Candidate drugs from the Malaria Box were diluted in DMSO from a starting concentration of 10 mM and placed into four 96-well plates. Compounds were diluted 100-fold into a 384-well black-bottom plates (Nunc/Thermo Fisher). An additional plate was made by diluting the previous 384-well plate 10-fold into another plate for a final concentration of 1 μM. Duplicate 384-well plates were made for each test concentration and amphotericin B was used as a batch control. Plates were allowed to incubate for 96 hours in humidity chambers and luminescence reads were conducted as previously mentioned in luminescence assay, dose-response determinations section. The Tecan EVO Freedom liquid handling system (Tecan US Inc., Durham, NC) was used to produce all drug assay plates and conduct all pipetting operations for this assay.

#### Robustness assessment (Z′).

Assay quality (Z′ factor) was determined based on the statistical test published by Zhang and others.^28^ To determine the Z′, half a plate of *L*. *major* amastigotes were exposed to amphotericin B, whereas the amastigotes on the other half of the plate were grown in the absence of any antileishmanials. Z′ was calculated as follows: Z′ = 1 − ([3σ_(+)_ + 3σ_(−)_]/μ_(+)_ − μ_(−)_), where μ_(+)_ and σ_(+)_ are the mean and standard deviation of the positive control, respectively; μ_(−)_ and σ_(−)_ are the mean and standard deviation of the negative control, respectively, and the denominator value is the absolute value of the difference in the positive and negative control means.^28^

#### Cytotoxicity assay.

An 3-(4,5-dimethylthiazol-2-yl)-2,5-diphenyltetrazolium bromide (MTT) cytotoxicity counter assay was used to test all compounds from the MMV Malaria Box for cytotoxicity. The 384-well MTT cytotoxicity assay is a modification of the MTT method described by Ferrari and others^29^ optimized for 384-well throughput. HepG2 target cells for this assay were cultured in DMEM media supplemented with 10% HIFBS (catalog no. 10438-034, Invitrogen). The HepG2 target cells for this assay were cultured as follows: HepG2 cells were cultured in complete Minimal Essential Medium (MEM; catalog no. 11090-099, Gibco-Invitrogen) prepared by supplementing MEM with 0.19% sodium bicarbonate (catalog no. 25080-094, Gibco-BRL), 10% HIFBS (catalog no. 16000-036, Gibco-Invitrogen, Grand Island, NY), 2 mM l-glutamine (catalog no. 25030-081, Gibco-Invitrogen), 0.1 mM MEM nonessential amino acids (catalog no. 11140-050, Gibco-Invitrogen), 0.009 mg/mL insulin (catalog no. I1882, Sigma), 1.76 mg/mL bovine serum albumin (catalog no. A1470, Sigma), 20 units/mL penicillin–streptomycin (catalog no. 15140-148, Gibco-Invitrogen), and 0.05 mg/mL gentamycin (catalog no. 15710-064, Gibco-Invitrogen). HepG2 cells cultured in complete MEM were first washed with 1× Hank's Balanced Salt Solution (catalog no. 14175-095, Invitrogen), trypsinized using a 0.25% trypsin/ethylenediaminetetraacetic acid solution (catalog no. 25200-106, Invitrogen), assessed for viability using trypan blue, and resuspended at 250,000 cells/mL. Drug plates were prepared with the Tecan EVO Freedom using sterile 96-well plates containing 12 duplicate 1.6-fold serial dilutions of each test compound suspended in DMSO. Of diluted test compound, 4.25 μL was then added to the 38.3 μL of media in each well providing a 10-fold final dilution of compound. Compounds were tested from a range of 57 to 10,000 ng/mL for all assays. The 4-azaindolo[2,1-b]quinazoline-6,12-dione was used as a toxicity control. After a 48-hour incubation period, 8 μL of a 1.5 mg/mL solution of MTT diluted in complete MEM media was added to each well. All plates were subsequently incubated in the dark for 1 hour at room temperature. After incubation, the media and drugs in each well was removed by shaking plate over sink, the plates were then left to dry in hood for 15 minutes. Next, 30 μL of isopropanol acidified by addition of hydrochloric acid at a final concentration of 0.36% was added to dissolve the formazan dye crystals created by reduction of MTT. Plates are put on a three-dimensional rotator for 15–30 minutes. Absorbance was determined in all wells using a Tecan icontrol 1.6 Infinite plate reader. The IC_50_ determinations were then generated for each toxicity dose–response test using GraphPad Prism (GraphPad Software Inc., San Diego, CA) using the nonlinear regression (sigmoidal dose–response/variable slope) equation.

## Results

### Robustness assessment of the intracellular *L*. *major* amastigote assay.

To assess the robustness of this high-throughput assay, we calculated the Z′ factor, signal-to-noise (S:N) ratio, and the signal-to-background (S:B) ratio derived from our initial development of an HTS assay using the luciferase-expressing *L*. *major* parasite. HTS assays that have a calculated Z′ factor value ≥ 0.5, an S:N ratio > 10, and an S:B ratio > 3 meet industry standards for HTS.^30^ Before implementation of a modular incubator chamber, we could not obtain Z′ values above 0.5 (mean Z′ = −0.56375). On incubating plates within a modular incubator chamber, Z′ values beyond the 0.5 threshold were consistently achieved, and we could reasonably use this assay in an HTS effort (Table 1). The luciferase-based amastigote macrophage assay's S:N and S:B ratios were calculated to reach well beyond industry minimum thresholds (mean S:N ratio of 180.8 and mean S:B ratio of 609.1 [Table 1]).

### Assay performance using antileishmanial standard drugs.

To further characterize the *L*. *major* dose–response assay, a series of antileishmanial standard drugs were examined. This panel included amphotericin B, miltefosine, paromomycin, pentamidine, pentostam, ketoconazole, and sitamaquine. The IC_50_ determinations are shown in Table 2 along with standard deviation values. The values listed are in broad agreement with published values against *L*. *major* obtained by other investigators.^14^,^31^–^35^

### Screening of the MMV Malaria Box collection.

To assess the utility of this new screening paradigm, we chose to assay a small collection of 400 compounds called the MMV Malaria Box. We chose to prescreen this collection at a 1 μM concentration, assaying each compound in duplicate wells against the luciferase-expressing intracellular *L*. *major* amastigote assay to determine each compound's percent growth inhibition. Compounds were selected for dose–response testing if the average percentage growth inhibition obtained was ≥ 60%. Of 400 compounds, 76 demonstrated intracellular *L. major* growth inhibitions of at least 60% in this prescreen, and dose–response assays were conducted as described in the section Materials and Methods. Out of these 76 compounds selected for dose–response testing, 14 compounds demonstrated IC_50_ determinations ranging from 15 to 477 nM, IC_50_ determinations were not obtained above 477 nM. A complete list of the potency data and structures for these 14 compounds is shown in Table 3. An in vitro cytotoxicity assay was conducted against all 14 hits, and the toxicity IC_50_ determinations for each compound are listed in Table 4.

## Discussion

We have developed a bioluminescent *L. major* (MHOM/SU/73/WR779) intracellular amastigote prescreen assay suitable for high-throughput drug screening. No data published yet demonstrates whether testing the promastigote, axenic amastigote, or the amastigote-macrophage form of the parasite yields the most relevant hits in vivo; however, what is clear from a number of published *Leishmania* drug screens is testing the amastigote-macrophage form of the parasite is the most stringent assay, which yields the lowest percentage of hits.^16^–^18^,^24^–^26^ This is likely due to the number of membranes a potential drug must cross to reach the intracellular amastigote-macrophage form of *Leishmania*. Many drugs have activity against the promastigote or axenic amastigote forms of the parasite, but these drugs may well fail to penetrate the outer membrane of the macrophage, the membrane-bound compartment of the parasitophorous vacuole (PV), and the membranes of the intracellular amastigote residing inside the PV.

Introducing the modular incubator chamber to our assay provided an essential component to creating an HTS assay with consistently positive Z′ factors, high S:N ratios, and high S:B ratios associated with robust performance. Without this modification, this assay functions well as an academic assay, but not as a robust, high-throughput screen.

Our screening of the MMV Malaria Box against *L. major* yielded a collection of 14 interesting hits with potency ranging from 15 to 477 nM. The collection delivered opportune starting points for lead optimization with a broad range of structural diversity as evidenced by the calculated properties (calculated logP = 1.9–7.4; polar surface area = 33–133; and rotatable bonds = 3–10).^36^ An in vitro therapeutic index, which was determined by ratio of HepG2 IC_50_/*L. major* amastigote-macrophage IC_50_, ranged from 4- to ≥ 400-fold. The 14 analogs that were active against *L. major* did not overlap specifically with any of the compounds observed to be active against *Leishmania infantum*.^13^ However, related analogs in the 1,3-dihydrobenzimidazol-2-imine class (MMV000444 and MMV000248) and quinazoline 2,4-diamino class (MMV006169 and MMV000963) did demonstrate activity against both the *L. major* and *L. infantum* strains.^13^

MMV666080 and MMV666023 were two compounds of interest. MMV666080 is an anticancer and matrix metalloproteinase inhibitor^37^–^39^ (*L. major* amastigote-macrophage IC_50_ = 15 nM), which was more potent and less toxic than the benchmark, positive control amphotericin B (IC_50_ = 27 nM). A possible subseries of substituted aminobenzimidazoles were identified to include MMV007564 (IC_50_ = 61 nM); MMV666023 (IC_50_ = 92 nM), both N1-benzylated; and MMV666607, an N1-H aminobenzimidazole with an IC_50_ = 134 nM.

Further profiling of potent antileishmanial compounds includes liver microsomal stability and permeability. If these hits meet selection criteria for stability and permeability, these compounds could be advanced to in vivo test models such as a suppression of mouse *L. major* lesion formation. Parasite burden and lesion size could be assessed in this type of experiment utilizing in vivo imaging technology,^25^ and drug blood levels could also be analyzed using blood spot technology.^4^,^40^ Further advancement of compounds with demonstrated antileishmanial efficacy in vivo involves testing candidate compounds for their ability to actually cure an established CL lesions caused by Old and New World *Leishmania* species in mice and hamsters.^4^ These in vivo lesion cure assays represent the most stringent test of a prospective CL compound outside of testing a compound in man.

In summary, we report the identification of 14 compounds out of the MMV Malaria Box with potency under 500 nM against *L. major* intracellular parasites. Our results confirm that compounds identified by MMV as antimalarial drugs can potentially be used outside the malaria field and form a foundation of drug development against CL. *Leishmania major* hits may have potential for use in the treatment of other forms of leishmaniasis.

## Figures and Tables

**Table 1:** Z′ factor, S:N, and S:B data calculated from prescreen assessments

Prescreen assay conditions	Z′ (mean, 95% CI)	S:N (mean, 95% CI)	S:B (mean, 95% CI)
Without modular incubator chamber	−0.56 (−17.654 to 0.407)	513.3 (0.682−5,286.46)	627.56 (8.53−2,746.55)
	*N* = 37	*N* = 37	*N* = 37
With modular incubator chamber	0.6 (0.51−0.76)	180.8 (40.6−272.8)	609.1 (162.1−954.5)
	*N* = 19	*N* = 19	*N* = 20

CI = confidence interval; S:B = signal-to-background ratio; S:N = signal-to-noise ratio.

Robust assays suitable for high-throughput screening have Z′ factors > 0.5, S:N ratios > 10, and S:B ratios > 3.

**Table 2:** IC_50_ determinations of standard antileishmanials against transgenic luciferase expressing intracellular *Leishmania major* amastigote-macrophage parasites

Compound	Geometric mean *L. major* IC_50_ (μM) IC_50_ ± SD (*n*)
Amphotericin B	0.027 ± 0.01 (32)
Miltefosine	0.55 ± 0.03 (5)
Paromomycin	0.78 ± 0.31 (3)
Pentamidine	3.42 ± 1.70 (5)
Pentostam	9.08 ± 0.35 (3)
Ketoconazole	19.85 ± 3.90 (5)
Sitamaquine	10.0 ± 0.37 (5)

**Table 3:** MMV Malaria Box *Leishmania major* hits

Compound ID	Structure	IUPAC name	MW (g/mole)	IC_50_ (nM)	*r*^*2*^
MMV666080	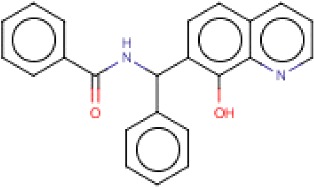	*N*-[(8-hydroxyquinolin-7-yl)-phenylmethyl]benzamide	354.4	14.9	0.97
MMV396693	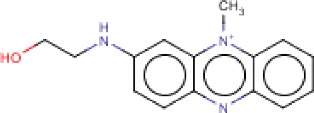	2-[(10-methylphenazin-10-ium-2-yl)amino]ethanol	254.31	53.4	0.97
MMV007564	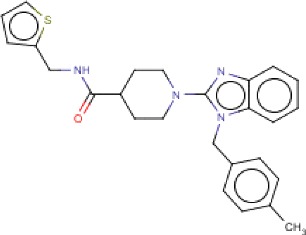	1-[1-[(4-methylphenyl)methyl]benzimidazol-2-yl]-*N*-(thiophen-2-ylmethyl)piperidine-4-carboxamide	444.59	60.8	0.95
MMV666023	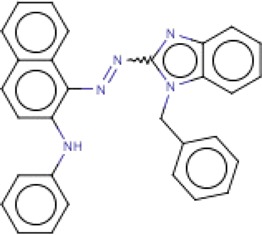	2-{2-[(4-nitrophenyl)methylidene]hydrazin-1-yl}-1*H*-1,3-benzodiazole	453.54	91.7	0.93
MMV007396	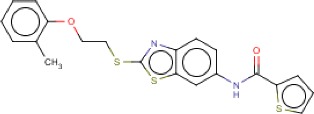	*N*-[2-[2-(2-methylphenoxy)ethylsulfanyl]-1,3-benzothiazol-6-yl]thiophene-2-carboxamide	426.57	119.8	0.94
MMV666607	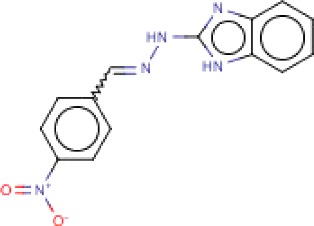	2-{2-[(4-nitrophenyl)methylidene]hydrazin-1-yl}-1*H*-1,3-benzodiazole	281.27	133.6	0.85
MMV007557	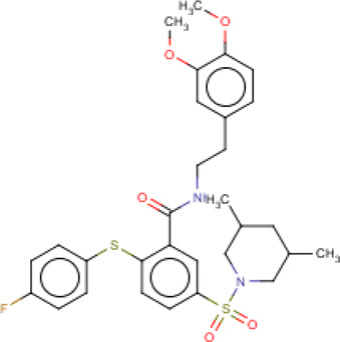	*N*-[2-(3,4-dimethoxyphenyl)ethyl]-5-(3,5-dimethylpiperidin-1-yl)sulfonyl-2-(4-fluorophenyl)sulfanylbenzamide	586.74	179.9	0.86
MMV667486	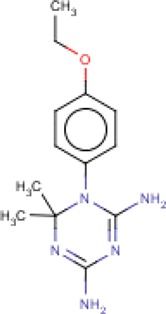	1-(4-ethoxyphenyl)-6,6-dimethyl-1,3,5-triazine-2,4-diamine	261.32	192.8	0.94
MMV008149	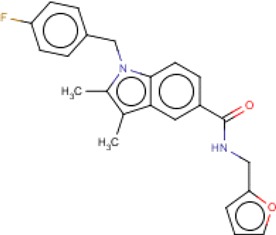	1-[(4-fluorophenyl)methyl]-*N*-(furan-2-ylmethyl)-2,3-dimethylindole-5-carboxamide	376.42	228.9	0.83
MMV000444	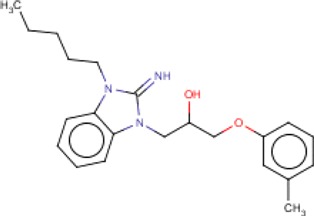	1-(2-imino-3-pentylbenzimidazol-1-yl)-3-(3-methylphenoxy)propan-2-ol	367.48	267.2	0.91
MMV666069	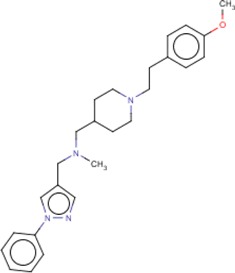	({1-[2-(4-methoxyphenyl)ethyl]piperidin-4-ylmethyl)(methyl)[(1-phenyl-1*H*-pyrazol-4-yl)methyl]amine	418.57	366.5	0.92
MMV007881	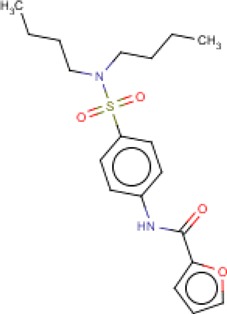	*N*-[4-(dibutylsulfamoyl)phenyl]furan-2-carboxamide	378.49	417.7	0.81
MMV006169	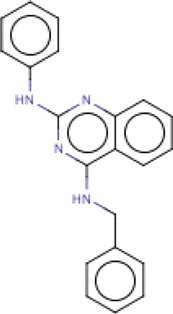	*N*-phenyl-*N*′-(phenylmethyl)quinazoline-2,4-diamine	326.39	466.6	0.90
MMV665827	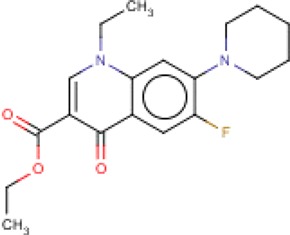	ethyl 1-ethyl-6-fluoro-4-oxo-7-piperidin-1-ylquinoline-3-carboxylate	356.40	477.7	0.81

IUPAC = International Union of Pure and Applied Chemistry; MMV = Medicines for Malaria Venture; MW = molecular weight.

**Table 4:** In vitro cytotoxicity assay for MMV Malaria Box *Leishmania major* hits

Compound ID	HepG2 cytotoxicity	
	IC_50_ (nM)	*r*^*2*^
MMV666080	> 6,060	N/A
MMV396693	5,662	0.91
MMV007564	> 6,060	N/A
MMV666023	1,305	0.96
MMV007396	3,497	0.92
MMV666607	> 6,060	N/A
MMV007557	> 6,060	N/A
MMV667486	> 6,060	N/A
MMV008149	3,257	0.86
MMV000444	4,126	0.92
MMV666069	5,817	0.96
MMV007881	5,603	0.85
MMV006169	5,658	0.80
MMV665827	1,720	0.93

MMV = Medicines for Malaria Venture; N/A = not available.
